# High Levels of Sedentary Time in Patients with COVID-19 after Hospitalisation

**DOI:** 10.3390/jcm11041110

**Published:** 2022-02-19

**Authors:** Bram M. A. van Bakel, Frederik M. A. van den Heuvel, Jacqueline L. Vos, Hajar Rotbi, Esmée A. Bakker, Robin Nijveldt, Dick H. J. Thijssen, Thijs M. H. Eijsvogels

**Affiliations:** 1 Department of Physiology, Radboud Institute for Health Sciences, Radboud University Medical Center, P.O. Box 9101, 6500 HB Nijmegen, The Netherlands; bram.vanbakel@radboudumc.nl (B.M.A.v.B.); hajar.rotbi@radboudumc.nl (H.R.); esmee.bakker@radboudumc.nl (E.A.B.); dick.thijssen@radboudumc.nl (D.H.J.T.); 2 Department of Cardiology, Radboud Institute for Health Sciences, Radboud University Medical Center, P.O. Box 9101, 6500 HB Nijmegen, The Netherlands; frederik.vandenheuvel@radboudumc.nl (F.M.A.v.d.H.); j.vos@radboudumc.nl (J.L.V.); robin.nijveldt@radboudumc.nl (R.N.); 3 Research Institute for Sports and Exercise Sciences, Liverpool John Moores University, Liverpool L3 5UX, UK

**Keywords:** COVID-19, SARS-CoV-2, coronavirus, physical activity, sedentary behaviour

## Abstract

Many patients with COVID-19 experience severe and even fatal disease. Survivors may have long-term health consequences, but data on physical activity and sedentary behaviour are scarce. Therefore, we objectively assessed physical activity (PA) patterns among post-hospitalised patients with COVID-19 and explored associations with patient characteristics, disease severity and cardiac dysfunction. We objectively assessed PA, sedentary behaviour and sleep duration for 24 h/day during 8 days at 3-6 months after COVID-19 hospitalisation. PA and sedentary time were compared across pre-defined subgroups based on patient and disease characteristics, cardiac biomarker release during hospitalisation, abnormal transthoracic echocardiogram at 3-6 months post-hospitalisation and persistence of symptoms post-discharge. PA and sedentary behaviour were assessed in 37 patients (60 ± 10 years old; 78% male). Patients spent 4.2 [3.2; 5.3] h/day light-intensity PA and 1.0 [0.8; 1.4] h/day moderate-to-vigorous intensity PA. Time spent sitting was 9.8 [8.7; 11.2] h/day, which was accumulated in 6 [5; 7] prolonged sitting bouts (≥30 min) and 41 [32; 48] short sitting bouts (<30 min). No differences in PA and sedentary behaviour were found across subgroups, but sleep duration was higher in patients with versus without persistent symptoms (9.1 vs. 8.3 h/day, *p* = 0.02). Taken together, high levels of sedentary time are common at 3–6 months after COVID-19 hospitalisation, whilst PA and sedentary behaviour are not impacted by patient or disease characteristics.

## 1. Introduction

Coronavirus disease 2019 (COVID-19) has a wide range of clinical manifestations, from mild symptoms with recovery at home, to severe illness and need for hospitalisation [1]. Emerging evidence exists that survivors of COVID-19 may suffer from long-term health consequences such as dyspnoea, fatigue and sleep difficulties [2]. A study from Italy found that 87% of post-hospitalised patients with COVID-19 experienced persistent symptoms at 60 days after onset [3]. A large Chinese cohort study among post-hospitalised patients with COVID-19 indicated that almost half of them still had at least one sequelae symptom at 12 months of follow-up [4]. Furthermore, these survivors of COVID-19 more often reported persistent mobility problems, had more pain or discomfort and suffered more frequently from anxiety or depression, compared to non-COVID-19 controls matched for age, sex and comorbidities [4]. A Dutch cohort study assessing clinical outcomes among patients with COVID-19 after 1 year of survival following intensive care unit treatment confirmed that persistent physical and mental symptoms are highly prevalent [5]. At the 1 year follow-up, more than a quarter of the patients reported mental health symptoms, whereas almost 75% experienced physical symptoms, predominantly due to joint symptoms, muscle weakness, general weakened condition and myalgia [5]. These findings indicate that persistent symptoms could have a detrimental impact on the patient’s daily life, which may ultimately lead to lower levels of habitual physical activity and fitness. High levels of sedentary time are known to increase the risk for the development of chronic diseases and long-term health outcomes [6,7]. Additionally, there is compelling evidence that physical inactivity prior to COVID-19 infection is strongly associated with severe COVID-19 outcomes [8]. Subsequently, becoming even more physically inactive is important from a re-infection point of view. However, objectively measured data on physical activity (PA) and sedentary behaviour after COVID-19-related hospitalisation, and how this is affected by markers of recovery, are scarce [9]. Most previous studies are based on self-reported data [10] and/or used tests of physical performance (i.e., 6-min walking test or 1-min sit-to-stand test) [11,12]. These outcomes do not provide insight into habitual PA and sedentary behaviour of survivors of COVID-19 in their daily life.

Therefore, the purpose of this study was to objectively assess PA, sedentary behaviour and sleep duration in patients with COVID-19 with moderate-to-severe illness that required hospitalisation, at three to six months after discharge. Additionally, we explored whether PA and sedentary time were impacted by patient and disease characteristics. We also explored echocardiography-assessed cardiac function and persistent symptoms post-discharge and its impact on PA and sedentary time. We hypothesised that post-hospitalised patients with COVID-19 will demonstrate high levels of sedentary time, especially those with comorbidities, a more severe COVID-19 course, cardiac dysfunction and persistent symptoms at 3–6 months post-discharge.

## 2. Materials and Methods

### 2.1. Population

Post-hospitalised patients with COVID-19 were invited for a single visit to objectively assess their PA patterns (i.e., moderate-to-vigorous PA (MVPA), light-intensity PA (LIPA), sedentary time and sitting bouts) and sleep duration. The study was part of a larger trial assessing the long-term outcomes after COVID-19 hospitalisation [13,14]. COVID-19 diagnosis was confirmed by polymerase chain reaction (PCR) testing of a nasopharyngeal sample and/or a non-enhanced low-dose CT thorax. Non-selected and consecutively admitted patients hospitalised at the COVID-19 nursing ward of the Radboud University Medical Center (Nijmegen, The Netherlands) were included between 1 April and 12 May 2020. Data on PA patterns were collected at 3 to 6 months after discharge. The study was approved by the local medical ethics committee (#2020-6765). All participants provided written informed consent. 

### 2.2. Measurements

PA patterns were assessed for 24 h/day during 8 consecutive days using the activPAL3^TM^ micro (PAL Technologies Ltd., Glasgow, UK) and participants completed a diary with wake and sleep times. The activPAL3^TM^ micro is a small device (25 × 45 × 5 mm) which is attached to the thigh of the patient using hypoallergenic and transparent tape (Tegaderm, 3M). To allow for continuous monitoring, the device is sealed with a nitrile sleeve and transparent tape for waterproof protection. The device combines a triaxial accelerometer with an inclinometer and has a sampling frequency of 20 Hz [15]. This enables the activPAL3^TM^ micro to accurately distinguish between sitting, standing and exercising [16]. After completion of the measurement, the device was sent back to our research institute by registered mail parcel. Raw data were analysed by a modified version of the script of Winkler et al. [17]. Activities were categorised as LIPA (metabolic equivalent of task (MET) score < 3) or MVPA (MET-score ≥ 3) [18]. Sedentary behaviour was defined as awake time spent at a MET-score ≤ 1.5 in a seated, reclined or lying posture [19]. Accumulation of sedentary time was examined by calculating the number of prolonged (≥30 min) and short (<30 min) sedentary bouts. 

Cardiac function was assessed by transthoracic echocardiography (TTE). All patients underwent a single TTE in supine position at the outpatient clinic at 3 to 6 months after discharge. We evaluated left and right ventricular function, diastolic left ventricular function and left ventricular global longitudinal strain (GLS). Valvular function or systolic pulmonary artery pressure were not extensively assessed. All TTEs were performed by experienced sonographers using the same ultrasound system (EPIQ, Philips Healthcare, Best, the Netherlands). The offline analysis was performed by a single, certified investigator using dedicated software (AGFA Enterprise Imaging Cardiology version 8.1.2, AGFA HealthCare, Mortsel, Belgium). GLS was measured using speckle tracking echocardiography on a three beats acquisition with a frame rate of >60 frames/sec. All measurements were performed according to the EACVI recommendations for cardiac chamber quantification [20]. Normal left and right ventricular volumes and function were defined following the international guidelines [20]: left ventricular ejection fraction of ≥52%, GLS of ≤−18%, tricuspid annular plane systolic excursion of ≥17 mm, right ventricular systolic excursion velocity of ≥10 cm/s, E/e′ ratio of ≤14, indexed left ventricular mass of ≤115 g/m^2^, indexed left ventricular end-diastolic dimension of ≤31 mm and right ventricular basal diameter of ≤42 mm If any of these criteria were not met, the TTE was classified as abnormal.

Patient characteristics (i.e., age; sex; body mass index (BMI), calculated by weight in kilograms divided by height in metres squared, extracted from electronic heath records upon hospital admission; smoking status), medical history (i.e., hypertension, diabetes mellitus, myocardial infarction, cerebrovascular disease, chronic renal failure and respiratory disease), laboratory results at admission, treatment (i.e., mechanical ventilation, intensive/medium care admission and duration of hospitalisation) and clinical outcomes (i.e., pulmonary embolism, acute kidney failure, acute heart failure, myocardial infarction, cerebrovascular event, ventricular arrhythmia, atrial fibrillation and myocarditis) were derived from electronic health records. Highly sensitive cardiac Troponin-T (hs-cTnT) and N-terminal pro-B-type natriuretic peptide (NT-proBNP) concentrations (Roche Diagnostics, Penzberg, Germany) were determined during hospitalisation. Abnormal concentrations of these cardiac biomarkers were defined as >14 ng/L and >300 pg/mL, respectively.

A nine-item online questionnaire was used to assess the presence of the following (persisting) COVID-19 symptoms at 6 months after discharge: dyspnoea (yes/no), chest pain (yes/no), peripheral oedema (yes/no) and fatigue (yes/no).

### 2.3. Statistical Analysis

Data were reported as number (%) for categorical variables, mean (±standard deviation (SD)) for normally distributed continuous variables and median [interquartile range (IQR)] for non-normally distributed continuous variables. We assessed the impact of (1) patient characteristics (i.e., age (≤ or >62 years), sex (female or male), BMI (≤ or >26.8 kg/m^2^) and comorbidities (hypertension, diabetes mellitus, myocardial infarction, cerebrovascular disease, chronic renal failure or respiratory disease); (2) disease characteristics (i.e., hospitalisation duration (≤ or >8 days), intensive care unit admission, occurrence of pulmonary embolism and abnormal cardiac biomarker concentrations); (3) cardiac dysfunction; and (4) persistent symptoms post-discharge, on PA patterns using a Student’s *t*-test or Mann–Whitney U test. Cut-off values of continuous variables were based on the median. All statistical tests were two-sided and significance was set at *p* < 0.05. Analyses were performed with IBM SPSS Statistics-25 (IBM Corp., Armonk, NY, USA).

## 3. Results

### 3.1. Study Population

**Figure 1:**
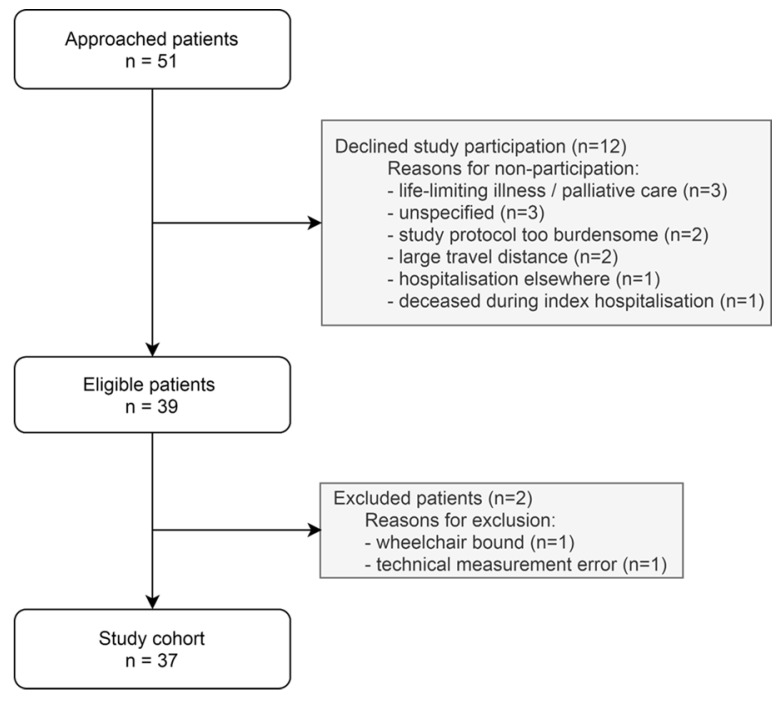
Flowchart of the study population.

We enrolled 37 post-hospitalised patients with COVID-19 in our study (Figure 1). Participants were 60 ± 10 years old, predominantly male (78%) with a median BMI of 26.8 [23.5, 29.6] kg/m^2^ (Table 1). Previously diagnosed hypertension (41%) was prevalent, followed by diabetes (16%), myocardial infarction (14%) and respiratory diseases (14%). COVID-19-related clinical characteristics and in-hospital complications are summarised in Table 1. The median hospital duration was 8 [7, 22] days and 13 (35%) patients were admitted to the intensive care unit. During hospitalisation, pulmonary embolism was diagnosed in 6 (16%) patients, hs-cTnT was elevated in 16 (43%) patients and 18 (49%) patients had elevated NT-proBNP levels. Transthoracic echocardiography at follow-up showed cardiac dysfunction in 9 (24%) post-hospitalised patients with COVID-19. Persistent symptoms (e.g., dyspnoea, chest pain, peripheral oedema and/or fatigue) were reported by 16 (43%) patients at 194 [185, 203] days post-hospitalisation.

### 3.2. Physical Activity and Sedentary Behaviour

**Figure 2:**
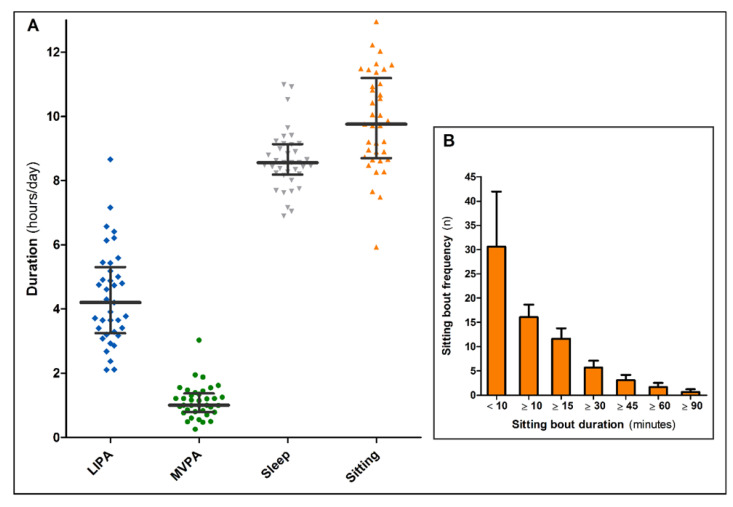
(**A**) objectively measured light-intensity physical activity (LIPA), moderate-to-vigorous intensity physical activity (MVPA), sleep duration and sedentary time in patients with COVID-19 at 125 [116, 132] days post-hospitalisation (box plots with median and interquartile range). (**B**) sitting bout frequency (mean + standard deviation) with a single bout duration ranging from <10 min up to ≥90 min.

Data on PA and sedentary time were collected at 125 [116, 132] days after discharge. Daily time spent in MVPA was 1.0 [0.8, 1.4] hours, while post-hospitalised patients with COVID-19 performed 4.2 [3.2; 5.3] hours of LIPA per day. Median sedentary time was 9.8 [8.7; 11.2] hours per day (Figure 2A), which was accumulated in 6 [5; 7] prolonged sitting bouts and 41 [32; 48] short sitting bouts (Figure 2B). Sleep duration was 8.6 [8.2; 9.1] hours per day (Figure 2A).

### 3.3. Physical Activity Patterns across Subgroups

Subgroup analysis indicated that PA levels and time spent sedentary were not impacted by patient characteristics, disease characteristics, cardiac dysfunction, nor by persistence of symptoms post-discharge. Sleep duration was higher in females (9.2 vs. 8.5 h/day, *p* = 0.03) and those who experienced persistent symptoms post-discharge (9.1 vs. 8.3 h/day, *p* = 0.02) (Table 2).

## 4. Discussion

We found that post-hospitalised patients with COVID-19 spent most of their time sedentary (±10 h/day), followed by light-intensity physical activity (±4 h/day) and moderate-to-vigorous physical activity (±1 h/day) at three to six months after hospital discharge. Sleep duration was ±9 h/day, which was slightly higher in women versus men and in patients with versus without persistent COVID-19 symptoms. In contrast to our hypothesis, PA and sedentary time were not impacted by patient and disease characteristics, nor by cardiac dysfunction and persistent symptoms post-discharge.

To the best of our knowledge, this is the first cohort study objectively assessing PA and sedentary time in post-hospitalised patients with COVID-19. Levels of sedentary time were remarkably lower (i.e., ±9 h/day) in a previous study among healthy controls of similar age, but with a higher prevalence of males (78% versus 62%) [21]. Nevertheless, it is unlikely that differences in sedentary behaviour between both cohorts were due to sex differences, as there is no evidence of sex-interaction effects in objectively measured total sedentary time in adults [22]. Therefore, post-hospitalised patients with COVID-19 seem to be more sedentary compared to healthy controls, but longitudinal cohort studies are warranted to confirm these cross-sectional observations. 

It is known that COVID-19 lockdown restrictions limit engagement in PA [23,24,25]. However, in the Netherlands, most lockdown measures were lifted at the time of data collection, which attenuates the likelihood that lockdown restrictions induced the high levels of sedentary time in post-hospitalised patients with COVID-19.

Current evidence suggests that long-term effects on habitual physical activity levels post-COVID-19 infection is of multifactorial nature and contributing factors are likely to vary across patients with COVID-19 [26]. These factors could include pathophysiological aspects such as systemic inflammation and (forced) physical inactivity due to decreased neural activation, fibre atrophy, muscle tissue necrosis and fibrosis, pulmonary fibrosis and alterations in blood flow and metabolic function [16]. Most likely, psychological factors also contribute to the high levels of sedentary time post-COVID-19 since many patients experience anxiety, depression and sleep difficulties persisting or presenting months after the initial infection [27]. Current evidence highlighted that these mental health outcomes are most prevalent among those patients that experience physical symptoms [28]. Taken together, time spent sedentary is high in post-hospitalised patients with COVID-19.

This observation is worrisome since it is associated with an attenuated immune system, loss of skeletal muscle mass, increased risk of incident cardiovascular and metabolic disorders and a lower quality of life [9]. Furthermore, the 1-year burden of cardiovascular disease incidence is substantially increased in patients that survived acute COVID-19 compared to non-COVID-19 controls [29]. Additionally, almost half of the post-hospitalised patients with COVID-19 in our cohort reported persistent symptoms post-discharge, especially due to dyspnoea, and 16% remained fatigued, which is in line with the literature [4]. These long-term health consequences require attention from health care professionals to continue physical reconditioning, also directly after hospital discharge.

An important strength of this study is the objective measurements of PA and sedentary behaviour. It contributes to knowledge regarding the largely uncertain long-term effects of COVID-19 hospitalisation and provides targets for future programs to (re-)activate these patients. The limitations of our study include the single-centre design and low sample size. Inherent in investigating consequences of the acute COVID-19 outbreak, assessments of PA patterns in the pre-COVID era, as well as longer term follow-up measurements, are lacking. Therefore, larger cohort studies with longitudinal follow-up using objective assessments of PA and sedentary behaviour are warranted to better understand the full spectrum and timeline of long-term consequences of COVID-19 hospitalisation on PA and sedentary behaviour levels. 

In conclusion, post-hospitalised patients with COVID-19 are sedentary for most of their time awake at 3–6 months post-hospitalisation. Looking at our results, promoting PA in post-hospitalised patients with COVID-19 warrants a stepwise approach, starting with reducing sedentary behaviour, followed by increasing LIPA and, thereafter, stimulating MVPA [30]. It was during the COVID-19 pandemic that many e-health solutions arose, and these initiatives appeared to be successful tools to promote PA [31]. Moreover, online- and home-based PA counselling seems feasible and scalable using e-health services. Since high levels of sedentary time in post-hospitalised patients with COVID-19 are not impacted by patient nor disease characteristics, a uniform approach for the (re-)activation of these patients seems indicated.

## Figures and Tables

**Table 1:** Patient characteristics.

Baseline Characteristics	*n* = 37	Missing Values
**Age (years)**	60 (±10)	0 (0%)
**Sex (male)**	29 (78%)	0 (0%)
**Body mass index (kg/m^2^)**	26.8 [23.5, 29.6]	0 (0%)
**Smoking**	2 (2%)	0 (0%)
**Comorbidities**		0 (0%)
Hypertension	15 (41%)	
Diabetes mellitus	6 (16%)	
Myocardial infarction	5 (14%)	
Heart failure	0 (0%)	
Cerebrovascular disease	1 (3%)	
Chronic renal failure (eGFR < 30 mL/min/1.73 m^2^ or dialysis)	1 (3%)	
Chronic respiratory disease (COPD or asthma)	5 (14%)	
**COVID-19 hospitalisation**		
**Laboratory findings at admission**		
Haemoglobin (mmol/L)	8.4 [7.8, 9.1]	0 (0%)
Leucocytes (10^9^/L)	6.9 [5.0, 9.9]	0 (0%)
C-reactive protein (mg/L)	85.0 [44.5, 179.0]	0 (0%)
Procalcitonin (µg/L)	0.20 [0.08, 1.08]	13 (35%)
eGFR (ml/min/1.73 m^2^)	79 [65, 90]	0 (0%)
Lactate (mmol/L)	1.3 [1.2, 1.7]	14 (38%)
pH	7.47 (±0.05)	6 (16%)
**Cardiac biomarker release during hospitalisation**		
hs-cTnT (ng/L)	13 [8, 21]	3 (8%)
hs-cTnT > 14 ng/L	16 (43%)	3 (8%)
NT-proBNP (pg/mL)	330 [77, 680]	2 (5%)
NT-proBNP > 300 pg/mL	18 (49%)	2 (5%)
**Treatment**		
Mechanical ventilation	11 (30%)	0 (0%)
Number of days	16 (±7)	
Prone ventilation	10 (27%)	
Intensive care unit admission	13 (35%)	0 (0%)
Medium care unit admission	1 (3%)	0 (0%)
Duration of hospitalisation (days)	8 [7, 22]	0 (0%)
**In-hospital complications**		0 (0%)
Pulmonary embolism	6 (16%)	
Acute kidney failure	2 (5%)	
Acute heart failure	3 (8%)	
Myocardial infarction (type 2)	1 (3%)	
CVA/TIA	1 (3%)	
Ventricular arrhythmia	0 (0%)	
Atrial fibrillation	3 (8%)	
Myocarditis	0 (0%)	
**Follow-up—clinical outcomes**		
Days of follow-up after discharge	194 [185, 203]	0 (0%)
Emergency department visit	1 (3%)	
Hospitalisation	3 (8%)	
Pulmonary embolism	1 (3%)	
Acute heart failure	0 (0%)	
Myocardial infarction	0 (0%)	
Atrial fibrillation	1 (3%)	
Echocardiographic parameters		0 (0%)
Normal LV and RV volumes and function ^a^	28 (76%)	
Persistent symptoms post-discharge ^b^		0 (0%)
Dyspnoea	10 (27%)	
Chest pain	3 (8%)	
Peripheral oedema	7 (19%)	
Fatigue	6 (16%)	

Data are presented as *n* (%), mean (±SD) or median [IQR]. CVA: cerebrovascular accident; hs-cTnT: highly sensitive cardiac Troponin-T; LV: left ventricular; NT-proBNP: N-terminal pro-B-type natriuretic peptide; RV: right ventricular; TIA: transient ischaemic attack. ^a^ Normal LV and RV volumes and function were defined as: LV ejection fraction of ≥52%, global longitudinal strain of ≤−18%, tricuspid annular plane systolic excursion of ≥17 mm, right ventricular systolic excursion velocity of ≥10 cm/s, E/e′ ratio (early mitral inflow velocity/mitral annular early diastolic velocity) of ≤14, indexed left ventricular mass of ≤115 g/m^2^, indexed LVEDd (left ventricular end-diastolic dimension) of ≤31 mm and RV (right ventricular) basal diameter of ≤42 mm. ^b^ Self-reported persistent symptoms post-discharge, assessed at 194 [185, 203] days of follow-up.

**Table 2:** Physical activity and sedentary time among subgroups of patients with COVID-19.

		*n* (%)		LIPA (h/Day)	MVPA (h/Day)	Sitting (h/Day)	Sleep (h/Day)
**Patient characteristics**							
**Age (years)**	≤62	19 (51%)		4.5 ± 1.6	1.2 [0.9; 1.4]	9.8 ± 1.8	8.5 ± 1.1
	>62	18 (49%)		4.2 ±1.3	1.0 [0.7; 1.4]	9.9 ± 1.3	8.7 ± 0.7
			*p*-value	0.52	0.43	0.94	0.49
**Sex**	Females	8 (22%)		4.5 ± 1.1	1.0 [0.8; 1.3]	9.3 ± 1.0	9.2 ± 1.0
	Males	29 (78%)		4.3 ± 1.6	1.1 [0.8; 1.5]	10.0 ± 1.6	8.5 ± 0.8
			*p*-value	0.73	0.70	0.22	**0.03**
**Body mass index (kg/m^2^)**	≤26.8	18 (49%)		4.7 ± 1.4	1.2 [0.8; 1.5]	9.7 ± 1.5	8.5 ± 0.9
	<26.8	19 (51%)		4.1 ± 1.6	1.0 [0.7; 1.3]	10.1 ± 1.6	8.8 ± 0.9
			*p*-value	0.25	0.27	0.45	0.35
**Comorbidity ^a^**	Yes	23 (62%)		4.5 ± 1.6	1.0 [0.8; 1.5]	9.6 ± 1.5	8.8 ± 1.0
	No	14 (38%)		4.2 ± 1.4	1.2 [0.9; 1.4]	10.3 ± 1.6	8.4 ± 0.6
			*p*-value	0.63	0.75	0.19	0.19
**Disease characteristics**							
**hs-cTnT (ng/L)**	≤14	18 (49%)		4.1 ± 1.2	1.0 [0.7; 1.4]	10.1 ± 1.1	8.7 ± 0.9
	>14	16 (43%)		4.5 ± 1.5	1.2 [1.0; 1.4]	9.7 ± 1.7	8.6 ± 1.1
			*p*-value	0.43	0.30	0.39	0.67
**NT-proBNP (pg/mL)**	≤300	17 (46%)		4.6 ± 1.6	1.0 [0.8; 1.2]	9.5 ± 1.5	8.9 ± 1.1
	>300	18 (49%)		4.1 ± 1.3	1.2 [0.8; 1.4]	10.1 ± 1.5	8.5 ± 0.6
			*p*-value	0.41	0.46	0.28	0.18
**Intensive care unit admission**	Yes	13 (35%)		4.2 ± 1.4	1.2 [0.7; 1.3]	9.9 ± 1.6	8.8 ± 1.3
	No	24 (65%)		4.5 ± 1.5	1.0 [0.8; 1.4]	9.8 ± 1.5	8.5 ± 0.6
			*p*-value	0.58	1.0	0.83	0.45
**Duration of hospitalisation (days)**	≤8	19 (51%)		4.7 ± 1.6	1.0 [0.8; 1.5]	9.7 ± 1.5	8.4 ± 0.7
	≥8	18 (49%)		4.1 ± 1.3	1.2 [0.8; 1.2]	10.0 ± 1.6	8.9 ± 1.1
			*p*-value	0.24	0.99	0.65	0.15
**Pulmonary embolism**	Yes	6 (16%)		3.7 ± 1.2	1.3 [0.9; 1.6]	10.5 ± 1.7	8.4 ± 1.3
	No	31 (84%)		4.5 ± 1.5	1.0 [0.8; 1.3]	9.7 ± 1.5	8.7 ± 0.8
			*p*-value	0.24	0.27	0.26	0.60
**Cardiac dysfunction post-discharge**							
**Normal TTE ^b^**	Yes	28 (76%)		4.5 ± 1.5	1.0 [0.8; 1.3]	9.8 ± 1.6	8.6 ± 1.0
	No	9 (24%)		3.9 ± 1.3	1.1 [0.8; 1.6]	10.0 ± 1.3	8.7 ± 0.5
			*p*-value	0.25	0.57	0.73	0.75
**Persistent symptoms post-discharge**							
**Persistent symptoms ^c^**	Yes	16 (43%)		4.5 ± 1.4	1.0 [0.8; 1.2]	9.4 ± 1.3	9.1 ± 1.1
	No	21 (57%)		4.3 ± 1.6	1.2 [0.8; 1.6]	10.2 ± 1.7	8.3 ± 0.6
			*p*-value	0.64	0.16	0.15	**0.02**

Data are presented as mean (±SD) or median [IQR]. Hs-cTnT: highly sensitive cardiac Troponin-T; LIPA: light-intensity physical activity; MVPA: moderate-to-vigorous physical activity; NT-proBNP: N-terminal pro-B-type natriuretic peptide; TTE: transthoracic echocardiogram. ^a^ Any comorbidity: hypertension, diabetes mellitus, myocardial infarction, cerebrovascular disease, chronic renal failure and/or chronic respiratory disease. ^b^ Normal TTE post-discharge was defined as: LV ejection fraction of ≥52%, global longitudinal strain of ≤−18%, tricuspid annular plane systolic excursion of ≥17 mm, right ventricular systolic excursion velocity of ≥10 cm/s, E/e′ ratio (early mitral inflow velocity/mitral annular early diastolic velocity) of ≤14, indexed left ventricular mass of ≤115 g/m^2^, indexed LVEDd (left ventricular end-diastolic dimension) of ≤31 mm and RV basal diameter of ≤42 mm. ^c^ Any self-reported persistent symptom post-discharge: dyspnoea, chest pain, peripheral oedema, fatigue.

## Data Availability

Original individual data are available upon reasonable request and can be obtained from the corresponding author.
